# Positive experience with treatment is associated with better surgical outcome in trapeziometacarpal osteoarthritis

**DOI:** 10.1177/1753193419851777

**Published:** 2019-06-01

**Authors:** Jonathan Tsehaie, Mark J. W. van der Oest, Ralph Poelstra, Ruud W. Selles, Reinier Feitz, Harm P. Slijper, Steven E. R. Hovius, Jarry T. Porsius, the Hand-Wrist Study Group

**Affiliations:** 1 Department of Plastic, Reconstructive and Hand Surgery, Erasmus MC Rotterdam, The Netherlands; 2 Hand and Wrist Surgery, Xpert Clinic, The Netherlands; 3 Department of Rehabilitation Medicine, Erasmus MC Rotterdam, The Netherlands

**Keywords:** Carpometacarpal, osteoarthritis, thumb, trapeziometacarpal, context, patient experience, PREMS, PROMS

## Abstract

The aim of this study was to investigate the association between patients’ experiences with trapeziometacarpal arthroplasty and treatment outcomes in terms of patient-reported outcome measures, grip and pinch strength. We included 233 patients who received a Weilby procedure for trapeziometacarpal osteoarthritis. Before surgery and 12 months after surgery, patients completed the Michigan Hand Outcomes Questionnaire, and their pinch and grip strengths were measured. At 3 months after surgery, a patient-reported experience measure was completed. Using regression analysis, significantly positive associations were found between the Michigan Hand questionnaire and the patient-reported experience measure, with the strongest significant associations being for patients’ experiences with information provision. No significant associations were found between the patients’ experience and strength outcomes. The results highlight the potential importance of positive experience with the treatment process to improve treatment outcomes in patients undergoing surgery for trapeziometacarpal osteoarthritis.

**Level of evidence:** IV

## Introduction

The context in which healthcare is delivered is an important part of a treatment, since the experience with healthcare delivery can contribute to treatment outcomes (Curran, 2007). The treatment context can be broadly defined as all aspects of the therapeutic context (e.g. treatment rationale, response to treatment) or the healthcare environment (e.g. quality of facilities, hygiene) that may affect patient perceptions across the continuum of care (Arnold et al., 2014; Connor-Greene, 1993; Wolf et al., 2014). When these aspects have an effect on treatment outcomes that cannot be attributed to the treatment itself, they are called ‘contextual effects’ (Miller and Kaptchuk, 2008; Moerman and Jonas, 2002). In many conditions, influencing the treatment context, for example by improving the communication between patient and clinician, can improve patient-reported health status (Di Blasi et al., 2001).

To measure these contextual aspects of a treatment, questionnaires are available that can reliably quantify the patient’s experience with the delivered healthcare: such questionnaires are called patient-reported experience measures (PREMs) (Manary et al., 2013). These questionnaires often focus on different domains of healthcare experience, such as communication with the physician or other healthcare providers, involvement of the patient in the decision-making, delivery of postoperative care and hygiene of the healthcare facilities. Together with patient-reported outcome measures (PROMs) and therapist recorded outcomes, such as strength and range of motion, PREMs are increasingly used as a measure of quality of care (Nilsson et al., 2016; Roland, 2004).

Observational studies have shown an association between healthcare experience (measured with PREMs) and PROMs in emergency surgery and elective surgery (Howe et al., 2017; Jones et al., 2017). For example, in hip replacement surgery, better experience with the healthcare process has been associated with better outcome as measured with the Oxford Hip Score (Black et al., 2014). Another study showed that general practitioners (GPs) who received training in communication and pain assessment before treatment for osteoarthritis had significantly better outcomes, that is, their patients experienced significantly less pain compared with patients whose GPs did not receive this training (Chassany et al., 2006). Moreover, in hand surgery, the empathy of the physician was the strongest driver of patient satisfaction, with 66% of the variation in patients’ satisfaction explained by the empathy of the physician (Menendez et al., 2015).

Although a relationship has been shown between expectations of treatment outcome and patient-reported outcome after treatment of trapeziometacarpal osteoarthritis (TMJOA) (Frouzakis et al., 2015), to our knowledge no study has investigated the effect of the experience of the delivered healthcare on outcome after treatment of TMJOA. The aim of this study was to investigate which aspects of the experience of healthcare delivery are associated with better treatment outcome after surgery for TMJOA in terms of both patient-reported outcomes and strength outcomes.

## Methods

### Study design and setting

This cohort study was carried out between February 2011 and April 2017 at Xpert Clinic in the Netherlands. Xpert Clinic is a specialized treatment centre for hand and wrist problems. It has 17 different locations, with 16 European Board-certified hand surgeons and over 50 hand therapists. The study was approved by the local institutional review board and written informed consent was obtained from all patients. Baseline characteristics of all patients (including age, gender, occupational status and hand dominance) were collected before the start of treatment.

Patients who underwent surgery for symptomatic TMJOA were included. During the study period, no non-certified hand surgeons or fellows did any of the surgical procedures. To include a homogenous group, patients who underwent a surgical treatment other than the Weilby (1988) procedure were excluded from the analysis. Also excluded were patients who did not fill in either the PROM questionnaires or the PREM questionnaires.

In the Weilby technique, the trapezium was removed and the flexor carpi radialis tendon was used to create a tendon interposition and ligament reconstruction. Postoperatively, patients had plaster cast immobilization for 3 to 14 days. Hand therapy was divided into two phases of 6 weeks. Phase one consisted of therapy to optimize the position of the thumb and to use a full thumb range of motion. In phase two, the patient practised the learned stability during daily activities and also improved thenar muscle strength (Van Uchelen et al., 2014). Delivery of treatment followed a standardized protocol to ensure that all patients received the same care.

### Outcome measures

To assess treatment outcome, patients were invited to fill in the Michigan Hand Questionnaire (MHQ, Dutch Language Version) before surgery and at 12 months after operation (Chung et al., 1998; Efanov et al., 2019; Marks et al., 2014; van der Giesen et al., 2008). The MHQ is a self-reported questionnaire with six domains (pain, aesthetics, hand function, performance of activities of daily living, work performance and satisfaction) and 37 items. It is scored from 0 (poorest function) to 100 (ideal function). For non-traumatic hand conditions, the minimal clinically important difference (MCID) for the total MHQ ranges from 9 to 13 points (London et al., 2014). Furthermore, all subdomains have excellent internal consistency, with Cronbach’s alpha ranging from 0.86 to 0.97 for the subscales (Chung et al., 1998). In this study, we decided to investigate the associations of both the total MHQ score as well as the different subscales of the MHQ with the PREMS subscales, since the MHQ is not a disease-specific questionnaire for TMJOA. As a result, some subscales of the MHQ are more relevant in TMJOA than others. For example, pain is known to be an important reason why patients visit the outpatient clinic (Menendez et al., 2015), while aesthetics rarely play a role. Consequently, we were interested in whether there were stronger associations between certain subscales of the MHQ.

We assessed the MHQ at 12 months because at 12 months patients are fully recovered from surgery and have completed the postoperative rehabilitation. Furthermore, we used the change in scores of the MHQ between baseline and 12 months to remove differences in patients regarding baseline MHQ.

To rate patients’ perceived experience with the healthcare provided, at 3 months patients completed a PREM questionnaire that is widely used in private practice clinics in the Netherlands (Poelstra et al., 2018). The PREM questionnaire consists of 25 items divided into six subscales to rate patients’ perceived experience. The six subscales were: quality of facilities (six items); physician communication and competence (six items); perioperative care (four items); postoperative care (four items); treatment information (three items); and general information (two items). Each item was graded on a 10-point ordinal scale, where 1 represents ‘very poor experience’ and 10 ‘excellent experience’. The full questionnaire is published in the study of Poelstra et al. (2018).

Using a Jamar-type hydraulic hand dynamometer, tip pinch and key pinch were measured by the hand therapist at baseline and at 12 months after operation. All strength measurements were recorded as the mean of three consecutive measurements (Mathiowetz et al., 1984) in accordance with the Dutch treatment guideline for TMJOA (Van Uchelen et al., 2014). The MCID was 0.33 kg for tip pinch and 0.84 kg for key pinch (Villafañe et al., 2017).

### Statistical methods

Paired *t*-tests were used to investigate whether the change in outcome measured in both PROMS and strength outcomes at 12 months after surgery was significant. Linear regression analysis was used to examine the univariable relationship between PREMS and the change in outcomes after surgery (PROMS and strength outcomes), which were reported as beta coefficients.

To examine the extent to which the variation in treatment outcomes between patients could be explained by the experience of the delivered healthcare, explained variance (R^2^) was calculated for treatment outcomes when all PREM subscales were entered simultaneously in a multiple linear regression model. To assess to what extent clustering influenced outcome due to the various surgeons and locations used in this study, we calculated intraclass correlations (ICCs). An ICC of 0.02 was found for the factor location and an ICC of 0.001 was found for the factor surgeon on outcome, indicating that the clustering attributable to different surgeons and locations was negligible. To prevent unnecessary complexity of the models, thereby reducing the interpretability of the results obtained, we therefore decided to not correct for which surgeon did the procedure or where the procedure took place using a mixed model regression analysis.

All analyses were done using R statistical computing, version 3.3.3 (R Development Core Team., 2019). For all tests, a *p-*value ≤0.05 was considered to be statistically significant.

## Results

**Figure 1.**
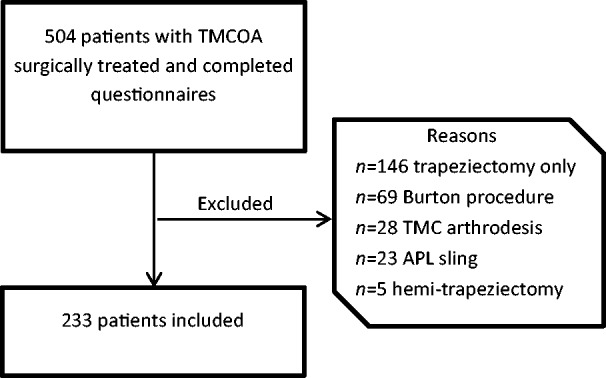
Flowchart showing the selection of patients and the reasons for exclusion. TMC OA: trapeziometacarpal osteoarthritis; APL: abductor pollicis longus; PROM: patient-reported outcome measure; PREM: patient-reported experience measure.

Between 2011 and 2017, 504 patients with TMJOA were treated surgically. After applying the exclusion criteria, 233 patients were included for analysis (Figure 1). The mean age of the patients was 59 years (SD 7.9; range 51–67) and 82% of the patients were female. Furthermore, 43% were either unemployed or retired and 45% had surgery on their dominant hand.

At 12 months after surgery, all improvements in the MHQ total and MHQ subscales were significant and clinically important (i.e. they exceeded the MCID described in Methods), except for the MHQ subscale ‘aesthetics’ (Table 1). Change in the key pinch strength at 12 months after surgery was not significant, whereas the improvement in tip pinch strength was significant but not clinically important (Table 1). In general, patients had very high satisfaction with the whole treatment experience, with all subscales of the PREMS scoring ≥8 on a 1–10 scale.

**Table 1.** Preoperative and postoperative outcome scores.

	Preoperative	Postoperative	*p*-value
PREM scores: median (IQR)			
Physician: communication and competence		8.3 (7.8–9.0)	
Perioperative care		8.5 (8.0–9.0)	
Postoperative care		8.4 (8.0–9.0)	
General information		8.2 (8.0–9.0)	
Treatment information		8.3 (7.7–9.0)	
Quality of facilities		8.4 (7.8–9.0)	
PROM scores: mean (SD)			
Total	48 (13)	69 (19)*	**<0.001**
General function	47 (16)	63 (18)*	**<0.001**
ADL	49 (21)	76 (22)*	**<0.001**
Pain	33 (13)	60 (23)*	**<0.001**
Aesthetics	79 (21)	85 (20)*	0.028
Satisfaction	28 (17)	65 (28)*	**<0.001**
Work	44 (23)	64 (28)*	**<0.001**
Hand strength			
Key pinch (kg)	4.4 (2)	4.8 (2)	0.51
Tip pinch (kg)	18.9 (9)	24.8 (9)*	**<0.001**

* IQR: interquartile range; ADL: activities of daily living; SD: standard deviation; PREM: patient-reported experience measures; PROM: patient-reported outcomes measures.

Significant *p*-values shown in bold font.

Regression analysis showed a positive association between PREM subscales and PROM subscales, with the ‘general information’ subscale of the PREM having the highest association with the change in PROM subscales (Table 2). Beta coefficients of the regression analysis are presented in Table 2 and show, for instance, that each 1-point improvement in PREM subscale general information (1–10) resulted in an 8.1-point increase on the MHQ satisfaction subscale (0–100). In contrast to the PROMS, no significant association was found between the PREM subscales and change in key pinch or tip pinch strength.

**Table 2.** Bivariable regression analysis.^1^

	Change in PROM							Change in TROM	
PREM	Total	General function	ADL	Pain	Aesthetics	Satisfaction	Work	Key pinch	Tip pinch
Physician communication and competence	4.0 (1.6 to 6.4) **(*p < *0.001)**	1.2 (−1.7 to 4.0) (*p = *0.185)	4.7 (1.1 to 8.2) **(*p = *0.008)**	5.5 (2.3 to 8.7) **(*p* < 0.001)**	3.5 (−0.1 to 7.0) (*p* = 0.080)	5.9 (1.8 to 9.9) **(*p = *0.005)**	5.4 (1.5 to 9.3) **(*p = *0.012)**	0.1 (−0.3 to 0.6) (*p = *0.671)	−0.3 (−2.3 to 1.8) (*p = *0.99)
Perioperative care	2.5 (0.0 to 5.0) (*p* = 0.083)	1.0 (−1.9 to 3.9) (*p = *0.501)	2.8 (−0.8 to 6.5) (*p = *0.177)	3.1 (−0.3 to 6.4) (*p = *0.100)	0.9 (−2.6 to 4.6) (*p = *0.659)	5.3 (1.1 to 9.4) **(*p = *0.038)**	3.4 (−0.6 to 7.4) (*p = *0.122)	0.2 (−0.2 to 0.6) (*p = *0.261)	−0.3 (−2.2 to1.5) (*p = *0.883)
Postoperative care	3.7 (1.5 to 5.9) **(*p = *0.001)**	1.7 (−0.8 to 4.3) (*p = *0.110)	4.6 (1.4 to 7.8) **(*p = *0.012)**	4.1 (1.1 to 7.0) **(*p* = 0.009)**	3.0 (−0.2 to 6.3) (*p = *0.053)	5.0 (1.3 to 8.7) **(*p* = 0.011)**	5.0 (1.4 to 8.5) (***p = *0.011)**	−0.2 (−0.5 to 0.2) (*p = *0.377)	−0.3 (−2.3 to 1.6) (*p = *0.732)
General information	4.8 (2.5 to 7.0) **(*p < *0.001)**	3.2 (0.5 to 5.9) **(*p = *0.018)**	5.7 (2.3 to 9.0) **(*p = *0.001)**	5.3 (2.2 to 8.3) **(*p* < 0.001)**	4.0 (0.6 to 7.3) **(*p = *0.012)**	8.1 (4.3 to 11.8) **(*p < *0.001)**	4.4 (0.7 to 8.1) **(*p = *0.014)**	0.1 (−0.3 to 0.6) (*p = *0.455)	−0.2 (−2.1 to 1.7) (*p = *0.780)
Treatment information	3.6 (1.3 to 5.9) **(*p = *0.005)**	0.7 (−2.0 to 3.3) (*p = *0.524)	3.0 (−0.3 to 6.4) (*p = *0.131)	3.9 (0.8 to 6.9) (***p = *0.025**)	4.7 (1.4 to 8.0) ** (*p = *0.006)**	6.2 (2.4 to 10.0) ** (*p = *0.005)**	3.8 (0.1 to 7.5) (*p = *0.078)	0.0 (−0.4 to 0.4) (*p = *0.926)	−0.6 (−2.5 to 1.3) (*p = *0.629)
Quality of facilities	4.5 (1.7 to 7.3) **(*p = *0.006)**	1.9 (−1.3 to 5.2) (*p = *0.133)	3.6 (−0.5 to 7.7) (*p = *0.143)	5.8 (2.1 to 9.5) (***p = *0.006)**	5.0 (0.9 to 9.1) ** (*p = *0.041)**	6.1 (1.4 to 10.8) ** (*p = *0.020)**	6.5 (2.0 to 11.0) ** (*p = *0.020)**	0.2 (−0.3 to 0.7) (*p = *0.490)	−0.3 (−2.5 to 1.9) (*p = *0.673)
Explained variance (R^2^)	8.4% ** (*p = *0.002)**	3.2% (*p = *0.196)	6.7% ** (*p = *0.01)**	7.1% ** (*p = *0.008)**	4.7% (*p = *0.084)	7.8% ** (*p = *0.010)**	5.0% (*p = *0.108)	4.4% (*p = *0.592)	0.0% (*p* = 0.996)

1 Bivariable regression analysis of the association between experience with the delivered healthcare (PREM) and outcome after surgery (PROM + strength outcomes), displayed as beta-coefficients (with 95% confidence intervals). The bottom row presents the results of the multiple regression analysis and shows how much of the variation in the subscales of the PROMS is explained by the PREM, when the PREM subscales are combined in one model to reflect the different subscales of the PROM and strength outcomes.

PREM: patient-reported experience measures; PROM: patient-reported outcomes measures; ADL: activities of daily living; TROM: therapist reported outcome measures.

Significant *p*-values shown in bold font.

Multiple regression analysis showed that, when combining all the individual PREM subscales into one model to match the PROM, the PREM subscales explained 3.2–8.4% of the variation in patient-reported outcome between patients (Table 2: bottom row). The PREM subscales had the strongest association with the total score of the MHQ, with 8.4% of the variance explained by the subscales of the PREM. Again, no associations were found between PREM subscales and change in key pinch or tip pinch strength.

## Discussion

The main aim of this study was to investigate which aspects of the experience of healthcare delivery are associated with treatment outcomes after surgery for trapeziometacarpal osteoarthritis of the thumb. It was found that patients who reported a more positive experience with the healthcare delivered had better self-reported outcomes in terms of pain and function. Patient experiences with the general information provided to patients and better postoperative care delivery were most strongly associated with a positive change in treatment outcomes. In contrast, no association was found between the experience of the care delivered and outcomes of hand strength. PREMs explained 3–8% of the variance in the change in patient-reported outcome.

Our findings are in line with similar studies, but with different patient populations. For example, in patients undergoing knee or hip replacement, Black et al. (2014) found that communication and trust in their doctor had the highest association with patient-reported outcome. We found similar results, with strong univariate associations between the physician’s communication and patient-reported outcome in terms of pain and satisfaction.

Since the role of treatment context on outcomes in hand surgery has not yet been thoroughly studied, it is difficult to compare our results with other studies. However, one study where the association between treatment context and treatment outcome after Dupuytren’s disease was examined showed that treatment context was also positively associated with PROMS (Poelstra et al., 2018). More specifically, it was found that the subscales ‘physician communication’, ‘postoperative care’ and ‘treatment information’ were most strongly associated with outcome. We found very similar results, with a strong association between the subscales ‘physician communication’ and ‘general information’ and patient-reported outcomes.

There are many reasons why the experience of healthcare delivery is associated with patient-reported outcomes. For example, we found that the general information provided on our website (https://www.xpertclinic.nl/handaandoeningen/duimbasis-artrose) and the brochure given to patients in the outpatient clinic had the highest associations with outcomes after surgical treatment for TMJOA. As we designed and produced a video for our website and a brochure showing the steps of surgery and what the entire treatment will consist of (including the postoperative rehabilitation process), patients may have felt they knew what to expect. This may have resulted in better compliance with the postoperative exercise regime, which may have led to better treatment outcomes. Another explanation is that providing adequate information on general treatment and good communication with the patient may lead to altered expectations of outcome. It is becoming clearer that treatment expectations are a cornerstone in context effects (Crow et al., 1999) and can be adjusted by discussing treatment beliefs (Laferton et al., 2016). The present study did not find a positive association between the treatment context and hand strength, possibly because no marked improvements in strength were seen after surgery.

Our study has both strengths and limitations. The main strength is the large sample population and the observational study design. Another strength is the relatively high level of generalizability, since our data were collected in daily clinical practice using the well-validated and tested MHQ. In addition, the collection of data took place in 17 outpatient clinics throughout the Netherlands, providing a representative sample of the population of patients with TMJOA. A limitation of the study is that the PREM questionnaire has not yet been thoroughly tested and may have omitted other important aspects of treatment context. Moreover, a limitation of the study is that the PREM questionnaire was filled in at 3 months and was potentially influenced by the pain and function experienced at this time. Since patients generally scored very highly on the PREM questionnaire, with all subscales of the PREMS scoring ≥8.0 on a 1–10 scale, a ceiling effect may have occurred. This could potentially lead to a decrease in variance and therefore a weaker association with the PROMS. For future research, a more sensitive PREM questionnaire is needed to assess the association with PROMS.

An important consideration is that it is impossible to know whether the associations are causal, that is, it remains unclear whether patients have a better outcome because of the better experience, or whether they have better experience because of a better outcome. Future studies with an appropriate design should investigate this. Moreover, we did not study how treatment context was associated with other outcomes, such as complications.

Owing to our study design, it is unclear whether there are factors that mediate the association between PREMs and PROMs. For example, patients who have more positive or optimistic expectations may have reported more positive experiences with the healthcare delivered, irrespective of the actual delivered care. Furthermore, it is becoming clearer that psychological factors play an important role in the level of perceived pain and disability caused by TMJOA. For example, one study found that patients who visited a doctor for complaints caused by TMJOA had a higher incidence of depression and had more catastrophic thinking compared with non-symptomatic patients with TMJOA (Becker et al., 2013). Another study found that anxiety and catastrophic thinking were correlated with perceived disability in patients with TMJOA (Lozano-Calderon et al., 2008).

In conclusion, the present study shows that experience with the delivered care of patients with TMJOA was positively associated with patient-reported outcomes, whereas there was no association between the experience with the delivered care and hand strength. This study highlights the potential importance of positive experiences with the treatment process for improving treatment outcomes in patients treated for TMJOA. Educating surgeons and other healthcare providers about such contextual effects may provide a valuable addition to their skills.

## Collaborators

Arjen Blomme, Berbel Sluijter, Corinne Schouten, Dirk- Jan van der Avoort, Erik Walbeehm, Gijs van Couwelaar, Guus Vermeulen, Hans de Schipper, Hans Temming, Jeroen van Uchelen, Luitzen de Boer, Nicoline de Haas, Oliver Zöphel, Sebastiaan Souer, Thybout Moojen, Xander Smit, Rob van Huis, Pierre-Yves Pennehouat, Karin Schoneveld, Yara van Kooij, Robbert Wouters, Paul Zagt, Folkert van Ewijk, Frederik Moussault, Rik van Houwelingen, Joris Veltkamp, Arenda te Velde, Alexandra Fink, Steven Hovius, Kim Spekreijse, Chao Zhou, Miguel Janssen, Stefanie Evers, Jak Dekker, Matijs de Jong, Jasper van Gestel, Marloes ter Stege, Menno Dekker, Roel Faber, Frank Santegoets, Monique Sieber-Rasch and Ton Gerritsen.

## References

[bibr1-1753193419851777] Arnold MH Finniss DG Kerridge I Medicine’s inconvenient truth: the placebo and nocebo effect. Intern Med J. 2014, 44: 398–405. 24754688 10.1111/imj.12380

[bibr2-1753193419851777] Becker SJE Briet JP Hageman MGJS Ring D Death, taxes, and trapeziometacarpal arthrosis hand. Clin Orthop Relat Res. 2013, 471: 3738–44. 23959907 10.1007/s11999-013-3243-9 PMC3825869

[bibr3-1753193419851777] Black N Varaganum M Hutchings A Relationship between patient reported experience (PREMs) and patient reported outcomes (PROMs) in elective surgery. BMJ Qual Saf. 2014, 23: 534–43. 10.1136/bmjqs-2013-002707 24508681

[bibr4-1753193419851777] Chassany O Boureau F Liard F, et al. Effects of training on general practitioners’ management of pain in osteoarthritis: a randomized multicenter study. J Rheumatol. 2006, 33: 1827–34. 16724375

[bibr5-1753193419851777] Chung KC Pillsbury MS Walter MR Hayward RA Reliability and validity testing of the Michigan Hand Outcome Questionnaire. J Hand Surg Am. 1998, 23: 575–87. 9708370 10.1016/S0363-5023(98)80042-7

[bibr6-1753193419851777] Connor-Greene PA The therapeutic context: preconditions for change in psychotherapy. Psychotherapy. 1993, 30: 375–82.

[bibr7-1753193419851777] Crow R Gage H Hampson S Hart J Kimber A Thomas H The role of expectancies in the placebo effect and their use in the delivery of health care: a systematic review. Health Technol Assess. 1999, 3: 1–96. 10448203

[bibr8-1753193419851777] Curran J The doctor, his patient and the illness. BMJ. 2007, 335: 941.

[bibr9-1753193419851777] Di Blasi Z Harkness E Ernst E Georgiou A Kleijnen J Influence of context effects on health outcomes: a systematic review. Lancet. 2001, 357: 757–62. 11253970 10.1016/s0140-6736(00)04169-6

[bibr10-1753193419851777] Efanov JI, Nguyen D-D, Izadpanah A, Danino MA, Harris P. A health utility assessment of trapeziectomy with ligament reconstruction and tendon interposition for thumb trapeziometacarpal osteoarthritis. J Hand Surg Eur. Epub ahead of print 16 April 2019. DOI: 10.1177/1753193419843850. 10.1177/1753193419843850 30987512

[bibr11-1753193419851777] Frouzakis R Herren DB Marks M Evaluation of expectations and expectation fulfillment in patients treated for trapeziometacarpal osteoarthritis. J Hand Surg Am. 2015, 40: 483–90. 25617218 10.1016/j.jhsa.2014.10.066

[bibr12-1753193419851777] Howe LC Goyer JP Crum AJ Harnessing the placebo effect: exploring the influence of physician characteristics on placebo response. Health Psychol. 2017, 36: 1074–82. 28277699 10.1037/hea0000499 PMC7608626

[bibr13-1753193419851777] Jones CH O’Neill S McLean KA Wigmore SJ Harrison EM Patient experience and overall satisfaction after emergency abdominal surgery. BMC Surg. 2017, 17. 10.1186/s12893-017-0271-5 PMC5494126 28668089

[bibr14-1753193419851777] Laferton JAC Auer CJ Shedden-Mora MC Moosdorf R Rief W Optimizing preoperative expectations in cardiac surgery patients is moderated by level of disability: the successful development of a brief psychological intervention. Psychol Heal Med. 2016, 21: 272–85. 10.1080/13548506.2015.1051063 26042657

[bibr15-1753193419851777] London DA Stepan JG Calfee RP Determining the Michigan Hand Outcomes Questionnaire minimal clinically important difference by means of three methods. Plast Reconstr Surg. 2014, 133: 616–25. 24572853 10.1097/PRS.0000000000000034

[bibr16-1753193419851777] Lozano-Calderon SA Souer JS Jupiter JB Ring D Psychological differences between patients that elect operative or nonoperative treatment for trapeziometacarpal joint arthrosis. Hand (NY). 2008, 3: 271–5. 10.1007/s11552-008-9098-y PMC2525886 18780109

[bibr17-1753193419851777] Manary MP Boulding W Staelin R Glickman SW The patient experience and health outcomes. N Engl J Med. 2013, 368: 201–3. 23268647 10.1056/NEJMp1211775

[bibr18-1753193419851777] Marks M Audigé L Herren DB Schindele S Nelissen RGHH Vliet Vlieland TPM Measurement properties of the German Michigan Hand Outcomes Questionnaire in patients with trapeziometacarpal osteoarthritis. Arthritis Care Res. 2014, 66: 245–52. 10.1002/acr.22124 23982906

[bibr19-1753193419851777] Mathiowetz V Weber K Volland G Kashman N Reliability and validity of grip and pinch strength evaluations. J Hand Surg Am. 1984, 9: 222–6. 6715829 10.1016/s0363-5023(84)80146-x

[bibr20-1753193419851777] Menendez ME Chen NC Mudgal CS Jupiter JB Ring D Physician empathy as a driver of hand surgery patient satisfaction. J Hand Surg Am. 2015, 40: 1860–5. 26231482 10.1016/j.jhsa.2015.06.105

[bibr21-1753193419851777] Miller FG Kaptchuk TJ The power of context: reconceptualizing the placebo effect. J R Soc Med. 2008, 101: 222–5. 18463276 10.1258/jrsm.2008.070466 PMC2376272

[bibr22-1753193419851777] Moerman DE Jonas WB Deconstructing the placebo effect and finding the meaning response. Ann Intern Med. 2002, 136: 471–6. 11900500 10.7326/0003-4819-136-6-200203190-00011

[bibr23-1753193419851777] Nilsson E Orwelius L Kristenson M Patient-reported outcomes in the Swedish National Quality Registers. J Intern Med. 2016, 279: 141–53. 26306802 10.1111/joim.12409

[bibr24-1753193419851777] Poelstra R Selles RW Slijper HP, et al. Better patients’ treatment experiences are associated with better postoperative results in Dupuytren’s disease. J Hand Surg Eur. 2018, 43: 848–54. 10.1177/1753193418780187 PMC6139991 29911473

[bibr25-1753193419851777] R Development Core Team. R: a language and environment for statistical computing. R Foundation for Statistical Computing, Vienna, Austria, 2019. http://www.r-project.org (accessed 25 April 2019).

[bibr26-1753193419851777] Roland M Linking physicians’ pay to the quality of care — a major experiment in the United Kingdom. N Engl J Med. 2004, 351: 1448–54. 15459308 10.1056/NEJMhpr041294

[bibr27-1753193419851777] van der Giesen FJ Nelissen RG Arendzen JH de Jong Z Wolterbeek R Vliet Vlieland TP Responsiveness of the Michigan Hand Outcomes Questionnaire-Dutch language version in patients with rheumatoid arthritis. Arch Phys Med Rehabil. 2008, 89: 1121–6. 18503809 10.1016/j.apmr.2007.10.033

[bibr28-1753193419851777] Van Uchelen J, Beumer A, Brink SM, Hoogvliet P, Moojen TM, Spaans AJ VG. Richtlijn conservatieve en chirurgische behandeling van primaire artrose van de duimbasis. Amsterdam: NVPC, NVVH, NOV, VRA, VvBN, 2014. https://www.nvpc.nl/uploads/stand/150416DOC-MB-Definitieve_richtlijn_Conservatieve_en_Chirurgische_behandeling_duimbasisartrose_28-10-2014_aangenomen_ALV_14_april_2015149.pdf (accessed 30 April 2019).

[bibr29-1753193419851777] Villafañe JH Valdes K Bertozzi L Negrini S Minimal clinically important difference of grip and pinch strength in women with thumb carpometacarpal osteoarthritis when compared to healthy subjects. Rehabil Nurs. 2017, 42: 139–45. 25557054 10.1002/rnj.196

[bibr30-1753193419851777] Weilby A Tendon interposition arthroplast of the first carpo-metacarpal joint. J Hand Surg Am. 1988, 13: 421–5. 10.1016/0266-7681_88_90171-4 3249143

[bibr31-1753193419851777] Wolf JA Niederhauser V Marshburn D Lavela SL Defining patient experience. Patient Exp J. 2014, 1: 7–19.

